# Presentation Variables Associated With the Development of Severe Post-obstructive Diuresis in Male Cats Following Relief of Urethral Obstruction

**DOI:** 10.3389/fvets.2022.783874

**Published:** 2022-04-05

**Authors:** Kelly M. Muller, Jamie M. Burkitt-Creedon, Steven E. Epstein

**Affiliations:** ^1^School of Veterinary Medicine, University of California, Davis, Davis, CA, United States; ^2^Department of Surgical and Radiological Sciences, School of Veterinary Medicine, University of California, Davis, Davis, CA, United States

**Keywords:** feline idiopathic cystitis, post-obstructive diuresis, hypovolemia [MesH], azotemia, hyperkalemia, acidemia, feline urethral obstruction, feline

## Abstract

**Background:**

Diuresis following relief of urethral obstruction is a potentially life-threatening complication of feline urethral obstruction. Evidence regarding the incidence of post-obstructive diuresis (POD) in cats is scarce. Establishing historical, physical examination, and initial clinicopathologic variables associated with risk for developing POD may better enable clinicians to direct treatment for this common feline emergency and to educate clients regarding financial expectations.

**Objectives:**

To report the incidence of POD in a large group of cats with urethral obstruction and determine whether select presenting physical examination or initial clinicopathologic variables may predict the onset or severity of POD.

**Methods:**

The records of 260 cats that were admitted to the University of California, Davis, Veterinary Medical Teaching Hospital for urethral obstruction were reviewed. Urine output after urethral catherization was categorized into no POD (urine output ≤ 2 mL/kg/h), mild-moderate POD (urine output > 2 but <5 mL/kg/h) and severe POD (urine output ≥ 5 mL/kg/h). Select presentation physical examination, venous acid-base, electrolyte, serum biochemistry, and urinalysis results were compared among the groups.

**Results:**

67.7% of cats experienced POD, and in 35% of cats it was categorized as severe. Evaluated historical and physical examination variables correlated with development of POD were lower body weight and, for severe POD, hypovolemia. Clinicopathologic variables associated with development of POD included acidemia, azotemia, hyperphosphatemia, hyperkalemia, hyponatremia, hypochloremia, hypocalcemia, hypermagnesemia, and hypoalbuminemia. Cats with severe POD were hospitalized a median of 1 day longer than those without POD.

**Conclusions:**

Results of the present study indicate that there are presentation variables associated with onset and severity of POD following relief of feline urethral obstruction.

## Introduction

Post-obstructive diuresis (POD) is a polyuric response sometimes seen following relief of urinary tract obstruction. In people severe POD has been reported to cause dehydration, electrolyte imbalances, hypovolemic shock, and death (1–5). Likewise, POD is a reported complication of feline urethral obstruction (UO) (6–8). It is likely that severe POD could lead to life-threatening hypovolemia in cats if left untreated, as it can in people. Two recent reviews of feline UO recommended that cats remain hospitalized with an indwelling urethral catheter following relief of UO to monitor for POD and to administer replacement IV fluids if it develops (7, 9). Identifying obstructed cats at the time of presentation that are at higher risk for POD could help clinicians to provide more accurate cost estimates and prognoses for clinical course to clients.

Two small studies have found that increased serum creatinine concentration is associated with development of POD following relief of UO in people (1, 2); one of these studies also reported that increased bicarbonate concentration was independently associated with development of POD (2). Two studies have investigated associations between presentation laboratory parameters and development of POD in cats following relief of UO (6, 8). Both studies compared cats with urine output (UOP) ≤ 2 mL/kg/h to those with UOP > 2 mL/kg/h and found that of all studied variables, only acidemia at presentation was predictive of the development of POD (6, 8). However, both of these studies followed cats for ≥ 48 h, during which time cats were treated with IV fluid therapy at rates equal to or greater than the cat's UOPs; thus, both studies had moderate to high risk of iatrogenic polyuria. Thus, the true incidence of POD in cats following relief of UO is unknown, and it is unclear whether any historical or presenting physical examination or clinicopathologic variables may predict its evolution or severity.

The objectives of the current investigation were to report the incidence of POD (UOP > 2 mL/kg/h) in a large group of cats following relief of UO; to report the incidence of severe POD (UOP ≥ 5 mL/kg/h) in these cats; and to determine whether any of the studied historical or presenting physical examination or clinicopathologic variables were associated with development of POD.

## Materials and Methods

The computerized medical records database for the University of California, Davis, Veterinary Medical Teaching Hospital was retrospectively searched for male or “sex unknown” cats treated for UO between January 1, 2002, and February 28, 2019, using the search terms “urinary,” “ureth^*^,” “obstruc^*^,” or “UO” in the presenting complaint, problem list, clinical diagnosis, or discharge summary fields. The resultant spreadsheets were merged and deduplicated. All data were recorded on standardized spreadsheets (Microsoft Excel, Version 16.43).

Cats were excluded if they were <1 year of age to ensure inclusion of only adults; if they were found to be female on physical examination; if they had traumatic, neurologic, or neoplastic disease preventing voiding of the bladder; if they had a pre-existing condition known to cause polyuria such as chronic kidney disease, diabetes mellitus, hyperthyroidism, or liver insufficiency or failure; if they had received medication (including steroids) expected to cause polyuria within 2 weeks prior to presentation or on day 1 of treatment for UO; if there was a prior medical report of polyuria; if there was evidence of a ruptured lower urinary tract during the study period; if they had a reported UO event in the prior 6 months; if they were discharged or euthanized immediately after relief of obstruction rather than being hospitalized; or if the cat had received therapy prior to presentation that could confound UOP measurement, such as fluid therapy in the 24 h prior to presentation. Cases were also excluded if the UOP could not be quantified based on medical records, if there were <4 h of UOP data available, or if medical records were missing.

Records were reviewed and the following presentation variables were recorded on a standardized spreadsheet: age, body weight, body condition score (BCS), presence of vomiting within the prior 48 h, duration of clinical signs recorded in 24 h time blocks, the presence of hypovolemia, and the number of days in hospital. A cat was considered hypovolemic if it had ≥3 abnormal perfusion parameters (mentation, mucous membrane color, capillary refill time, heart rate, pulse quality, subjective extremity temperature) and at least one of the following: (a) hypovolemia listed as a clinical diagnosis or problem in the medical record or (b) received an IV fluid bolus (defined as ≥ 10 mL/kg isotonic crystalloid) within 1 h of presentation. Urine output was recorded as described below. Laboratory variables recorded for analysis were presentation point-of-care venous acid-base and electrolyte panel (ABL 705, ABL 800, ABL 815; Radiometer Medical A/S, Copenhagen, Denmark); serum biochemistry panel (Chemistry analyzer, Hitachi 917; and Chemistry analyzer, Hitachi c501; both Roche Diagnostics, Indianapolis, IN); and urine specific gravity, pH, and glucose and ketone presence (Urine Test Strips, Chemstrip 10UA; and Urinalysis Analyzer, Urisys 1800; both Roche Diagnostics, Indianapolis, IN). Where the same variable was measured on both the point-of-care analyzer and on the reference laboratory chemistry analyzer, values from the point-of-care analyzer were used for statistical analysis. Due to the retrospective nature of this study, not all variables were available in the medical record for every cat, and not all cats underwent the same diagnostic battery.

### Ensuring That Initial Bladder Volume Was Not Included in Hourly UOP

It is our standard practice to empty the bladder entirely prior to attaching the sterile urine collection system (UCS) to the indwelling catheter. However, if UOP was recorded within 1 h of catheterization, that urine volume was considered to represent urine from the obstructed bladder and was not factored into UOP calculations; instead, the next collection period was considered the cat's first. To ensure retrospectively that the initial (“blocked”) urinary bladder volume was not considered part of the cat's post-obstructive UOP, twelve hours of UOP data were recorded. Cases were manually reviewed for consistency of UOP from first to subsequent UCS emptying periods over the full 12 h. When visual inspection of the spreadsheet suggested that the cat's first recorded UOP included urine from the obstructed bladder, that cat was excluded from analysis. The final 8 h of UOP data were not considered for any other purpose in this study.

### Urine Quantification and Definitions of POD

The time at which the UCS was attached was considered Time 0. UOP was generally measured every 4 h during hospitalization per hospital protocol. Only the first 4 h time block of urine collection was used to define a cat's status regarding presence of POD. Any cat with UOP > 2 mL/kg/h during this first 4 h time block was considered to have POD. Two mL/kg/h was chosen as the cutoff for diuresis based on other investigators' use of the same cutoff in this species (6, 8) and additional authors describing the upper limit of normal feline UOP as 2 mL/kg/h (10, 11). Cats with UOP > 2 but <5 mL/kg/h were considered to have mild-moderate POD, while those ≥5 mL/kg/h were considered to have severe POD.

### Statistical Methods

Data were analyzed for normality by visual inspection of histograms and the Kolmogorov-Smirnov test using commercially available software (IBM SPSS Statistics for Mac version 27.0, Armonk, NY). Continuous data are presented as mean ± standard deviation or median (Interquartile range, IQR: 25 to 75th percentile) as appropriate. Categorical data are presented as number (percentage). As the majority of the data were not normally distributed, a Kruskal Wallis ANOVA was used to compare continuous data between the three groups with a Dunn-Bonferroni *post-hoc* test. Categorical data were compared using a Fisher's exact test. A *P* < 0.05 was considered significant. For laboratory values that showed a significant difference between UOP groups, a Pearson's correlation coefficient was calculated. A priori the strength of the correlation was defined as 0 to 0.10 = negligible, 0.11 to 0.39 = weak, 0.40 to 0.69 = moderate, 0.70 to 0.89 = strong, and ≥0.90 = very strong.

## Results

The computerized search yielded 1,005 cases. Four hundred thirty-two cases were removed because the cat was found not to be obstructed upon record review; an additional 313 cases met one or more exclusion criterion (Supplementary Table 1). Consequently, 260 cases were included in the study.

All cats were male and were classified as intact (*N* = 13; 5%), cryptorchid (*N* = 1; 0.4%), or castrated (*N* = 246; 94.6%). Breeds included domestic shorthair cats (*N* = 160), domestic medium hair cats (44), domestic longhair cats (24), Siamese (9), Mixed (8), Persian (4), Maine Coon (4), Bengal (2), and one of each of the following: Bombay, Himalayan, Manx, Ragdoll, Somali. The median age was 5 years (IQR, 3–7 years). Median body weight was 6.0 kg (IQR, 5.2–6.8 kg).

The overall incidence of POD was 67.7% (*N* = 176/260). The incidence of mild-moderate POD was 32.7% (*N* = 85/260) and the incidence of severe POD was 35% (*N* = 91/260). 32.3% (*N* = 84/260) of cats did not experience POD. Cats without POD had a median UOP of 1.3 mL/kg/h (range, 0.16–2.0 mL/kg/h); cats with mild-moderate POD had a median UOP of 3.1 mL/kg/h (range, 2.1–4.9 mL/kg/h); and cats with severe POD had a median UOP of 7.7 mL/kg/h (range, 5.0–26.9 mL/kg/h).

Cats without POD (*N* = 84) weighed more (mean 6.4 ± 1.2 kg) than cats with severe POD (N = 91; mean 5.9 ± 1.5 kg; *P* = 0.029). There were no significant differences between the weight of cats with mild-moderate POD (*N* = 85, median 5.9 kg; IQR, 4.8–6.7 kg) and either other group.

A small number of cats (*N* = 19/260; 7.3%) presented hypovolemic. More cats that developed severe POD were hypovolemic at presentation (13/91; 14.2%) than cats that did not develop POD (2/84; 2.4%; *P* = 0.006). There was no difference between cats that experienced mild-moderate POD that were hypovolemic (4/85; 4.7%) compared to cats that did not develop POD (2/84; 2.4%; *P* = 0.68). Thus, the presence of hypovolemia in this group of cats presenting for UO has an estimated positive predictive value of 89.5% for development of POD.

Cats without POD (*N* = 82) had a shorter median duration of hospitalization (median 3.0 days; IQR, 2.0–3.3 days) than cats with severe POD (N = 91; median 4.0 days; IQR, 3.0–5.0 days; *P* < 0.001). There were no significant differences between the duration of hospitalization of cats with mild-moderate POD (N = 85, median 3.0 days; IQR, 3.0–4.0 days) and either other group.

Select results of the venous acid-base and electrolyte panel, serum biochemistry panel, and urinalysis are shown in Table 1. There was no difference among any of the groups in age; BCS; recent history of vomiting; blood PCV; blood glucose or lactate concentrations; blood PvCO_2_ or PvO_2_; serum activities of ALT, AST, or ALP; serum globulin, cholesterol, or total bilirubin concentrations; urine pH; or ketonuria.

**Table 1:** Presentation venous acid-base and electrolyte panel, serum biochemistry panel, and urinalysis values in adult male cats with urethral obstruction that did not develop post-obstructive diuresis (POD; *N* = 84/260), those that developed mild-to-moderate POD (*N* = 85/260), and those that developed severe POD (*N* = 91/260) following urethral catheterization.

		**Mild-moderate**	**Severe POD**		***Rho***
**Variable**	**No POD (UOP ≤2 mL/kg/h)**	**POD (UOP > 2 but <5 mL/kr/h)**	**(UOP ≥5 mL/kg/h)**	**Pearson's Rho**	***P-Value***
Albumin (g/dL)	3.7 (3.3–3.9; *N* = 48)	3.5 (2.8–3.7; *N* = 45)	3.1 ± 0.57 (*N* = 56)^*^	−0.35	<0.0001
Urine Specific Gravity (refractometer)	1.041 (1.031–1.045; *N* = 67)	1.033 ± 0.011 (*N* = 67)	1.022 ± 0.098 (*N* = 61) ^*^ 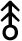	−0.55	<0.0001
Blood Urea Nitrogen (mg/dL)	28 (22–48; *N* = 48)	66 (27–131; *N* = 45) ¶	133 ± 81.6 (*N* = 56) ^*^ 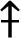	0.60	<0.0001
Creatinine (mg/dL)	1.5 (1.3–2.4; *N* = 48)	3.8 (1.5–8.1; *N* = 45) ‡	8.5 ± 5.5 (*N* = 56) ^*^ 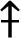	0.57	<0.0001
Phosphorus (mg/dL)	4.5 ± 1.2 (*N* = 48)	5.2 (4.2–7.3; *N* = 45) †	7.7 (5.0 - 11.3; *N* = 55) ^*^ 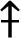	0.54	<0.0001
Sodium (mEq/L)	153 (151–154; *N* = 53)	151 (149–154; *N* = 64)	147 ± 5.52 (*N* = 69) ^*^ 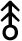	−0.13	0.1339
Potassium (mEq/L)	3.7 (3.4–4.0; N = 54)	4.2 (3.6–5.3; *N* = 64) ‡	6.4 (4.2 - 8.9; *N* = 71) ^*^ 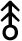	0.49	<0.0001
Chloride (mEq/L)	122 ± 4.38 (*N* = 52)	120 (115–123; *N* = 59) †	115 (107–119; *N* = 68) ^*^ 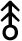	−0.31	0.0004
Ionized Calcium (mmol/L)	1.28 (1.17–1.34; N = 51)	1.22 (1.03–1.30; *N* = 64) †	1.07 ± 0.199 (*N* = 71) ^*^ 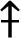	−0.47	<0.0001
Magnesium (mg/dL)	2.4 ± 0.17 (*N* = 22)	2.5 (2.3–2.6; *N* = 24)	2.7 (2.3 - 3.6; *N* = 31) §	0.45	<0.0001
pH	7.305 ± 0.072 (*N* = 41)	7.268 (7.209–7.315; *N* = 53) †	7.182 ± 0.124 (*N* = 61) ^*^ 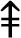	−0.51	<0.0001
Bicarbonate (mmol/L)	17.6 ± 2.8 (*N* = 36)	17.3 (14.5–18.8; *N* = 47) ¶	13.6 (11.1–16.6; *N* = 58) ^*^ 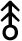	−0.48	<0.0001
Base Deficit (mmol/L)	−7.3 (-10.1 to−4.7; *N* = 41)	−8.5 (-13.1 to−6.5; *N* = 53) ¶	−12.8 ± 5.12 (*N* = 62) ^*^ 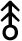	−0.51	<0.0001

*Variables are represented as mean ± SD when normally distributed and as median (25th to 75th percentile) when not normally distributed. N, number of cats; †, P-Value for Comparison of No POD vs. Mild-Moderate POD ≤ 0.05. ‡, P-Value for Comparison of No POD vs. Mild-Moderate POD ≤ 0.01. ¶, P-Value for Comparison of No POD vs. Mild-Moderate POD ≤ 0.001. §, P-Value for Comparison of No POD vs. Severe POD ≤ 0.01. ^*^, P-Value for Comparison of No POD vs. Severe POD ≤ 0.001. 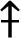, P-Value for Comparison of Mild-Moderate POD vs. Severe POD ≤ 0.05. 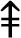, P-Value for Comparison of Mild-Moderate POD vs. Severe POD ≤ 0.01. 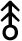, P-Value for Comparison of Mild-Moderate POD vs. Severe POD ≤ 0.001*.

## Discussion

This study is the first to document the incidence of severe POD (UOP ≥ 5 mL/kg/h) in a large group of cats following relief of UO. The results of the present study indicate that the presence of hypovolemia at presentation may predict the development of POD. Furthermore, many clinicopathologic variables measured at presentation (blood pH, bicarbonate concentration and base deficit; blood sodium, potassium, chloride, and ionized calcium concentrations; and serum albumin, urea, creatinine, phosphorus, and magnesium concentrations) are associated with development of POD following relief of UO. Finally, cats with severe POD were hospitalized significantly longer than those that did not develop POD.

Post-obstructive diuresis may be due to accumulation of excess diuretic solutes such as urea during the period of UO, though the pathophysiology is not fully understood (12). There is evidence in rats that POD may be due to decreased aquaporin-2 activity, which would reduce collecting duct responsiveness to antidiuretic hormone and cause diuresis (13). Other evidence suggests that POD may be caused by progressive reduction in medullary concentration gradient or downregulation of sodium transporters in the ascending loop of Henle (14). The pathophysiology may involve a combination of mechanisms.

The overall incidence of POD in this population was 67.7%. Two studies in people found the incidence of POD to be 30–63% (1, 2); it is unknown whether the underlying pathophysiology is the same between the 2 species. The incidence in the current study is similar to the incidence reported in two other feline studies, which found POD in 46–74% of cats following relief of UO (6, 8).

Increasing severity of azotemia, hyperphosphatemia, and hyperkalemia, and a lower USG at presentation were correlated with presence and severity of POD in this study. Higher serum creatinine concentration at presentation has been associated with evolution of POD in people (1, 2). In the current population of cats, increased urea, creatinine, potassium, and phosphorus concentrations were likely due to decreased glomerular filtration rate (GFR), which may indicate that more severe kidney injury may lead to more severe POD. Damaged renal tubules are unable to reabsorb solutes, which results in larger volumes of more dilute urine. Additionally, accumulated plasma urea may act as an osmotic diuretic, and it may reverse the renal vasoconstriction typically seen with obstructive uropathies (12). In accordance with these concepts, USG was negatively correlated with presence and severity of POD in this population of cats.

In the present study, there was a moderate correlation between presentation acid-base variables and severity of POD. Cats with severe POD were more acidemic, had lower bicarbonate concentration, and more negative base deficits than those that did not develop POD. The high anion gap can be explained by retention of organic acids by the urinary tract (15). Moreover, the more severe the cat's azotemia, acidemia, and electrolyte abnormalities, the more likely the cat was to develop POD, and POD severity correlated with degree of these clinicopathologic derangements.

There was an association between hypovolemia and development of severe POD. Cats that presented hypovolemic likely had a longer duration of obstruction and were thus more severely clinically affected. Hypovolemia causing poor perfusion was likely another reason for acidemia in cats with severe POD. However, blood lactate concentrations were not found to be significantly correlated with POD in the present study. There is some evidence that azotemic animals have lower plasma lactate concentrations due to decreased cellular production and increased lactate consumption, which may have impacted this population (16).

Severity of hypoalbuminemia was negatively correlated with the occurrence and severity of POD in the present study. Albumin is a negative acute phase protein and hypoalbuminemia may have been a marker of severity and duration of inflammation in these cats (17, 18). It is also possible that prolonged urinary tract obstruction led to glomerular injury, causing albumin to be lost into the urine in some cases. Unfortunately, meaningful information about proteinuria could not be determined in this study. Urethral obstruction has been associated with proteinuria in people (19). Cats with kidney injury due to UO may also develop proteinuria.

Urethral obstruction has been reported to cause ionized hypocalcemia in cats (20, 21). In the present study, ionized hypocalcemia at presentation was moderately correlated with severity of POD. Hypothesized pathophysiology of hypocalcemia in critically ill animals includes calcium binding by excess phosphorus, parathyroid hormone resistance, and acid-base alterations (21). The acidemia seen in many cats in this study may have led to underestimation of ionized hypocalcemia that would have been more severe at normal pH. No cats had clinical signs of ionized hypocalcemia, and it is difficult to know the clinical relevance of this finding.

Hypermagnesemia at presentation was correlated with onset and severity of POD. Hypermagnesemia can occur secondary to reduced GFR from acute kidney injury (22). Clinical signs of hypermagnesemia are rare in cats (23) and re-establishment of GFR following relief of UO may be adequate to correct it.

Cats with higher body weight were less likely to develop POD. This finding is likely due to how UOP is calculated, in mL/kg/h. A higher body weight decreases the hourly UOP for the same volume of urine produced. This finding is consistent with Balsa et al. (24) who found that cats with lower body weight had greater severity of POD following relief of ureteral obstruction. Additionally, cats with a higher body weight would likely have received higher IV fluid rates since these rates are calculated based on body weight. Despite likely higher IV fluid rates, these cats were less likely to develop POD. This finding supports the authors' belief that the POD observed in this study was unlikely to be iatrogenic.

Interestingly, vomiting within 48 h of presentation was not associated with POD. It had been hypothesized that vomiting may be an indicator of prolonged disease and would therefore be associated with more severe kidney injury, azotemia, and POD. Vomiting may have been underreported if clients did not observe or report it, or it may not have been included in the medical record. This finding is in contrast to Balsa et al. (24) who found that vomiting was associated with POD after relief of ureteral obstruction.

Both studies that previously searched for presentation variables that might predict POD in cats following relief of UO compared cats with UOP ≤ 2 mL/kg/h to those with UOP > 2 mL/kg/h (6, 8). Our definition of severe POD as UOP ≥ 5 mL/kg/h (2.5 x the physiologic upper limit) was an estimate based on the degree of diuresis that the authors suspect could meaningfully hamper a cat's ability to maintain itself by eating and drinking alone. At UOP ≥ 5 mL/kg/h, a cat weighing this study's median 6.0 kg would need to drink 720 mL per 24 h. This degree of polydipsia may prevent the cat from performing basic functions such as sleeping. While the 5 mL/kg/h cutoff value is somewhat arbitrary, we believed it may distinguish cats that could be more safely discharged immediately after urethral catheterization from those that may benefit from hospitalization.

Similar to our study, the prior two found that acidemia predicted POD (6, 8); however, those studies found no further associations between POD and other variables studied. There are likely a few reasons for the differences between our findings and theirs. Francis et al. (6) found that the majority of POD occurred >18 h after relief of obstruction, when fluid therapy during hospitalization was more likely to have impacted urine production. This confounder, in combination with their small sample size of 32 cats, may have decreased their chances of finding associations between these variables and POD. Frohlich et al. (8) determined that the POD in their study was at least partially iatrogenic because their fluid protocol involved administration of a higher IV fluid rate in the current time period than urine volume produced in the prior time period, which would increase a cat's UOP incrementally as IV fluid rates increased. Additionally, the Frohlich et al. (8) study included only 57 cats. The present study includes 260 cats and UOP was analyzed for only the first 4 h following relief of obstruction such that fluid therapy was less likely to impact UOP. In a study in rats undergoing bilateral ureteral ligation, POD occurred within 3 h of relief of obstruction (12); thus, we believe that a 4 h study period would have included most cases that experienced POD while reducing the impact of fluid therapy.

This study has several limitations, many due to its retrospective design. First, some cats may have had undiagnosed or unrecorded comorbid conditions that affected UOP. Second, there is no way to check the recorded values for accuracy and some medical records were incomplete, which led to exclusion of many cases and missing values in some included cats. Baseline (pre-obstruction) measures of kidney function were unavailable for most cats, so conditions such as chronic kidney disease may not be recognized; however, cats with UO tend to be younger (median age in this population was 5 years) than cats with other chronic illnesses. It is impossible to be certain that initial fluid therapy did not lead to higher early urine output in some cases; however, cats that received fluid boluses in our emergency room were likely given these fluids due to appropriate clinical decision making based on physical examination characteristics indicating hypovolemia, significant dehydration, or both. Thus, the authors suspect that the association between hypovolemia at presentation and evolution of severe POD in cats following relief of UO is reliable.

Results of the present study suggest that there are historical and presenting physical examination and clinicopathologic variables that predict the onset and severity of POD. Cats with azotemia, hyperkalemia, hyperphosphatemia, acidemia, ionized hypocalcemia, hypoalbuminemia, hypermagnesemia, or hypovolemia at presentation may be at higher risk of developing post-obstructive diuresis. Clinicians and clients should be aware of the possibility of longer hospitalization and higher cost of care for cats with these problems at presentation. Prospective evaluation of the predictive value of these variables and clinical outcomes is warranted.

## Data Availability Statement

The raw data supporting the conclusions of this article will be made available by the authors, without undue reservation.

## Ethics Statement

Ethical review and approval was not required for the animal study because retrospective study design. Written informed consent for participation was not obtained from the owners because retrospective study design.

## Author Contributions

KM: study development, literature search, data collection, and manuscript preparation. JB-C: study development, literature search, data analysis, manuscript preparation, and manuscript editing. SE: study development, statistical and data analysis, and manuscript editing. All authors contributed to the article and approved the submitted version.

## Funding

Online publication of this manuscript was funded by the Center for Companion Animal Health and the Open Access Fund of the University Library, both at the University of California, Davis.

## Conflict of Interest

The authors declare that the research was conducted in the absence of any commercial or financial relationships that could be construed as a potential conflict of interest.

## Publisher's Note

All claims expressed in this article are solely those of the authors and do not necessarily represent those of their affiliated organizations, or those of the publisher, the editors and the reviewers. Any product that may be evaluated in this article, or claim that may be made by its manufacturer, is not guaranteed or endorsed by the publisher.
